# Nailfold microscopy in adult-onset dermatomyositis in association with myositis antibodies

**DOI:** 10.1007/s00403-024-03521-z

**Published:** 2024-11-20

**Authors:** Elizabeth M. Flatley, Dina Collins, Tess M. Lukowiak, Jason H. Miller

**Affiliations:** 1 Robert Wood Johnson Medical School, Rutgers University, 125 Paterson St, New Brunswick, NJ USA; 2 Center for Dermatology, Rutgers Robert Wood Johnson Medical School, Somerset, NJ USA

**Keywords:** Dermatomyositis, Antibodies, Capillaroscopy, Dermoscopy, Nailfold

## Abstract

Dermatomyositis (DM) is an immune mediated inflammatory disease classically associated with muscular and cutaneous involvement. Existing studies have suggested characteristic nailfold findings may be observed in DM, indicating a potential role for nailfold microscopic examination in the diagnosis of DM. To that end, we performed a systematic review of literature pertaining to nailfold microscopic, capillaroscopic, and dermoscopic findings observed in patients with DM, with a secondary review of the association of nailfold microscopic findings with myositis-specific antibody (MSA) and myositis-associated antibody (MAA) status. Thirty-seven papers, representing 346 patients, met inclusion criteria. The most prevalent nailfold findings were evidence of increased vascular diameter (64.5%, *n* = 223) and decreased vascularity (57.5%, *n* = 199). Scleroderma-spectrum pattern and microhemorrhage or hemorrhage were both observed in 156 (45.1%) patients. Fifty-one patients had nailfold capillaroscopic findings reported in direct association with antibody positivity and demonstrated a range of nailfold findings, preliminarily suggesting associations between antibody-status and capillaroscopic findings. The results of our study align with existing data indicating the scleroderma-spectrum pattern and the pattern’s composite features are prevalent in patients with DM. Our review further demonstrates a variety of nailfold capillaroscopic findings are found in patients positive for MSAs and MAAs.

**Supplementary Information:**

The online version contains supplementary material available at 10.1007/s00403-024-03521-z.

## Introduction

Dermatomyositis (DM) is an immune mediated inflammatory disease associated with classical cutaneous and muscular manifestations, with a subset of patients presenting with amyopathic DM [1]. Several myositis-specific antibodies (MSAs) and myositis-associated antibodies (MAAs) have been identified, with associations established between these antibodies and characteristic phenotypic manifestations of DM [2]. Accurate diagnosis of DM is essential given its known association with malignancy and systemic disease, with the potential for long-term negative outcomes if left untreated. However, diagnosis may prove difficult due to the array of cutaneous features classically associated with DM and the potential for variable patient presentation [1, 3]. Existing studies have suggested characteristic microscopic nailfold findings may be observed in DM, indicating a potential role for nailfold capillaroscopy in the diagnosis of DM [4]. The diagnostic utility of microscopic examination of nailfolds is reinforced by the non-invasive and inexpensive nature of capillaroscopy and dermoscopy, allowing for in-vivo visualization of nailfold morphology [5, 6].

Accordingly, we performed a systematic review of the literature pertaining to the microscopic findings of the nailfold observed in patients with DM. This review serves as an updated and expanded synthesis of the literature, focusing on the nailfold features observed via microscopic examination in patients with adult-onset DM, with the aim of strengthening the existing evidence [7]. Included studies were secondarily reviewed to explore the association of nailfold capillaroscopic findings with myositis-specific antibody (MSA) and myositis-associated antibody (MAA) status. To our knowledge, no previous systematic review has examined the association of MSAs and MAAs with nailfold capillaroscopic findings. Recognition of the nailfold capillaroscopic features seen in DM may aid in diagnosis, potentially decreasing diagnostic ambiguity and time to diagnosis [8].

## Methods

### Literature review

We used Covidence software to search electronic databases (MEDLINE, Scopus, and Embase) using the search terms: “dermatomyositis”, “polymyositis dermatomyositis”, “myositis”, “dermato-myositis”, “dermatomycomositis”, “dermatopolymyositis”, ““adult type dermatomyositis”, “dermatomyositides”,“dermatomyositis”, “dermatopolymyositis”, “polymyositis arthropathica”, “unverricht-wagner syndrome”, “wagner-unverricht syndrome”, “wagner-unverricht”, or “wegner hepp. unverrricht disease”, in combination with the search terms: “microscopy”, “epiluminescence microscopy”, “trichoscopy”, “onychoscopy”, “dermatoscopy”, or “dermoscopy”. The search terms: “childhood type dermatomyositis”, “juvenile dermatomyositis”, or “juvenile myositis” were used to identify articles that contained both adult patients and juvenile patients, but only the results from adult patients were collected for this study. Databases were searched in March 2024. No limitation dates were set for study inclusion.

### Article selection

**Fig. 1:**
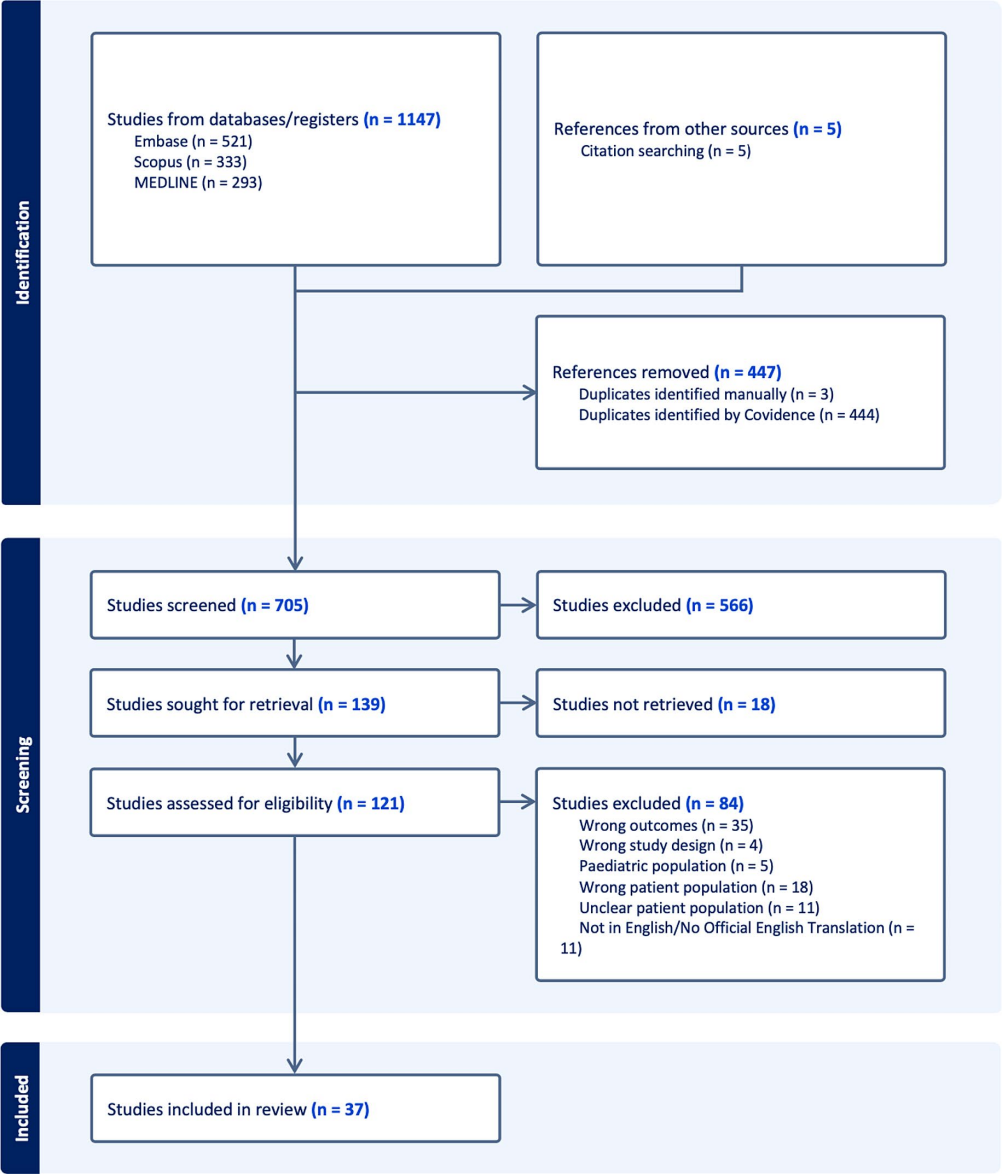
Article selection

Papers pertaining to the microscopic, capillaroscopic, and/or microscopic features of the nailfold in patients with adult-onset DM were included. No time restriction was established for paper inclusion. All papers not written in English, animal studies, and papers not addressing nailfold capillaroscopic/microscopic features of DM were excluded. Papers addressing the nailfold findings of patients with diseases and syndromes other than DM, patients with juvenile DM (JDM), or patients with dermatomyositis overlap disease were excluded, unless these papers also included patients with adult-onset DM and clearly distinguished the nailfold findings of these patients from the nailfold findings of JDM, overlap syndrome, or non-DM conditions. The corresponding author of all papers viable for inclusion in which the patient ages were not stated were contacted to confirm that all included patients > 18 years of age prior to inclusion in this review. Finally, papers that did not report the gross number or percentage of patients with nailfold findings, including studies with any findings reported as a median or mean number or studies with findings remarked upon as present with no indication of the number of patients with each finding, were excluded. Articles were sequentially screened based on title, abstract, then full text, based on the aforementioned criteria. Two independent reviewers (EF, DC) screened and reviewed studies (Fig. 1).

### Data extraction

Each included article was reviewed and the following datapoints were extracted, if available: article title/DOI, the continent and country in which the study was conducted, the total number of study participants, total number of study participants with DM, MSAs (anti-Mi-2 autoantibody, anti-SAE1/2 autoantibody, anti-jo-1 autoantibody, anti-OJ-autoantibody, anti-EJ-autoantibody, anti-KS-autoantibody, anti-Zo-autoantibody, anti-Ha/YRS autoantibody, anti-PL12 autoantibody, anti-PL-1 autoantibody, anti-MDA5 autoantibody, Anti-TIF-1γ autoantibody, and anti-NXP2 autoantibody) and MAAs (anti-PM-Scl, anti-Ku, anti-RNP, anti-SSA/Ro, and anti-SSB/La antibodies). Additionally, the gross number and/or percentage of patients with all reported nailfold microscopic findings were extracted.

Online Resource 1 (Supplementary Table 1) lists all nailfold findings from each reported study. For the purposes of this study, only findings identified via microscopic examination were marked as present. Any findings observed clinically, rather than via nailfold microscopic, capillaroscopic, or dermoscopic examination, were not marked as present. Finally, for studies that reported nailfold findings at multiple follow-up points, the highest total number or percentage of patients with findings was the number reported.

If authors reported a pattern of findings, consisting of *≥* 1 associated features, only that pattern was marked as present and counted within the overall percentage of features, unless the number of patients with the individual features composing that pattern was reported separately within the results. If authors reported ‘enlarged’, ‘large’, or ‘widening’ vessels, these were marked distinctly from ‘dilated’ or ‘giant/megacapillaries’ unless authors provided definitions of ‘enlarged’ ‘large’ or ‘widening’ that met the following criteria: dilated capillaries (a diameter > 20 µm and < 50 µm) or mega/giant capillaries (a diameter > 50 µm) [8].

Finally, in this paper, all microscopic, capillaroscopic, and dermoscopic findings of the nailfold are reported as ‘microscopic’ findings.

## Results

### Nailfold features

One thousand, one hundred, and forty-seven studies were initially identified, with five additional studies subsequently identified via citation searching. Following the elimination of duplicate studies, 705 studies were selected for screening. Ultimately, thirty-seven papers describing the microscopic nailfold findings of 346 patients met inclusion criteria.

The most prevalent nailfold finding was evidence of increased vascular diameter (64.5%, *n* = 223), including dilated vessels (*n* = 96, 27.7%), megacapillaries/giant vessels (*n* = 94, 27.2%), large/enlarged/widening vessels (*n* = 29, 8.4%), and telangiectasias (*n* = 4, 1.2%). The 2nd most prevalent finding was decreased vascularity, seen in 199 (57.5%) of included individuals, including the presence of avascular areas (*n* = 121, 35.0%) and loss of capillaries (*n* = 78, 22.5%).

An equal number of patients were found to have evidence of a scleroderma-spectrum pattern, classically defined as a combination of features including capillary enlargement, capillary bed deformation, capillary loss, capillary budding, and/or microhemorrhage [9]. The scleroderma-spectrum pattern, encompassing the early, active, and late scleroderma patterns, as well as scleroderma-dermatomyositis patterns, scleroderma-like patterns, author-defined scleroderma patterns, and ‘unspecified’ scleroderma patterns, was seen in 156 (45.1%) patients. Likewise, microhemorrhage/hemorrhage was found via microscopic examination to be present in 156 (45.1%) of included patients.

Finally, 108 individuals, representing 31.2% of included patients, had evidence of disturbed or disorganized capillaries or architecture. The remainder of nailfold microscopic findings were observed in less than 100 patients. All features identified and the total number of patients with each feature are reported in Table 1 [10–47].

**Table 1:** Prevalence of microscopic nailfold findings in patients with DM

Microscopic Nailfold Findings		Number of Patients (Percentage of All DM Patients [*n* = 346])
Increase in Vessel Diameter	**Total**	223 (64.5%)
	Dilated Vessels	96 (27.7%)
	Megacapillaries/Giant Vessels	94 (27.2%)
	Large/Enlarged/Widening Vessels	29 (8.4%%)
	Telangiectasias	4 (1.2%)
Decreased Vascularity	**Total**	199 (57.5%)
	Avascular Area	121 (35.0%)
	Loss of Capillaries	78 (22.5%)
Scleroderma Spectrum Patterns	**Total**	156 (45.1%)
	Unspecified Scleroderma Pattern	78 (22.5%)
	Early Scleroderma Pattern	10 (2.9%)
	Active Scleroderma Pattern	5 (1.4%)
	Late Scleroderma Pattern	2 (0.6%)
	Early and Active Pattern	1 (0.3%)
	Author-Defined Scleroderma Pattern	8 (2.3%)
	Scleroderma-Like Pattern	19 (4.3%)
	Scleroderma-Dermatomyositis Pattern	45 (13.0%)
Microhemorrhage/Hemorrhage		156 (45.1%)
Disturbed Distribution/Disorganized Capillaries/Disorganized Architecture		108 (31.2%)
Neovascularization	**Total**	73 (16.5%)
	Bushy Capillaries	64 (18.5%)
	Budding (Bushy OR Neovascularization)	4 (1.2%)
	Neoangiogenesis	3 (0.9%)
Unspecified Abnormal Shape		65 (18.8%)
Elongation		63 (18.2%)
Cuticular Changes	**Total**	47 (13.6%)
	Ragged Cuticles	42 (12.1%)
	Cuticular Dystrophy	1 (0.3%)
	Cuticular Hypertrophy	1 (0.3%)
	Cuticular Hemosiderin-Containing Deposits	1 (0.3%)
Normal/Hairpin/No Findings		36 (10.4%)
Nonspecific Findings		26 (7.5%)
Periungal Erythema		26 (7.5%)
Crisscross capillaries		20 (5.8%)
Abnormal Blood Flow	**Total**	17 (4.9%)
	Stasis	14 (4.0%)
	Thrombosis	2 (0.6%)
	Decreased Blood Flow	1 (0.3%)
Tortuous Capillaries		14 (4.0%)
Haem Deposits		12 (3.5%)
Subpapillary Plexus		8 (2.3%)
Dilation and Bleeding Pattern		8 (2.3%)
Edema		4 (1.2%)
Dotted Vessels		2 (0.6%)
Findings Observed in Singular Patients	Bizarre Capillary Loops	1 (0.3%)
	Capillary Pigmentation/Hyperpigmentation	1 (0.3%)
	Variant and Simple Raynaud’s	1 (0.3%)
	Y-shaped Vessel Formation	1 (0.3%)
	Extravasates	1 (0.3%)
	Striking Shape Heterogeneity	1 (0.3%)
	Vascular Rearrangement	1 (0.3%)
	Dendritic Tortuosity	1 (0.3%)
	Proximal Nail Fold Dystrophy	1 (0.3%)
	Nailfold Hyperkeratosis	1 (0.3%)

### Association with myositis-specific and myositis-associated antibodies

Fifty-one patients had nailfold microscopic findings reported in direct association with positive antibody status [10–22]. Of these 51, 31 were positive for anti-MDA-5 autoantibody, 10 for anti-TIF1-γ autoantibody, 6 for anti-NXP-2 autoantibody, and 2 for anto-Jo-1 autoantibody. Positivity for anti-Ro-52 autoantibody and anti-Mi2 autoantibody was observed in one patient each. No patients were positive for anti-SAE1/2, EJ, KS, ZO, HA, YRS, PL-12, PL-1, PM-SCL, KU, RNP, and SSB/LA antibodies, or did not have nailfold microscopic findings reported in connection with positivity for these autoantibodies.

Of the 31 patients who were anti-MDA-5 positive, 15 had scleroderma-spectrum pattern abnormalities (1 with early and active, 14 with scleroderma-spectrum abnormalities unspecified in association with MDA-5 positivity). Additionally, 13 demonstrated microhemorrhage or hemorrhage, 13 had vessel dilation or enlargement, and 8 showed areas of capillary loss. All other observed features were seen in single patients and did not occur across multiple anti-MDA-5 positive patients.

Amongst the 10 anti-TIF1-γ autoantibody positive patients, 8 were found to have nailfold scleroderma-spectrum abnormalities. Additional findings observed in a single patient were enlarged capillaries, evidence of giant capillaries, and hemorrhages.

All 6 patients positive for anti-NXP2 autoantibody demonstrated increases in vessel diameter and loss of capillaries. Four of the anti-NXP2 positive patients, included within a single study, were found to have evidence of hemorrhages, defined by the authors as “more than 2 punctate hemorrhages per finger or confluent hemorrhage areas” [22]. Finally, both anti-Jo-1 autoantibody positive patients demonstrated bushy/ramified capillaries, microhemorrhage/hemorrhage, decreased vascularity, and mega/giant capillaries but did not otherwise demonstrate overlap in features.

For the patients who were positive for a MSA or MAA antibody and had nailfold findings reported in association with their autoantibody status, only a single patient was positive, limiting the generalizability of findings. All nailfold findings found for MSA and MAA-positive patients are summarized in Table 2.

**Table 2:** Microscopic nailfold findings of MSA/MAA-Positive patients

Antibody Positivity	Citation	Number of Patients	Nailfold Microscopic Findings (*N*/Gross Number of Patients in Study/Report)
**Anti-MDA-5**	**Sugimoto 2023**	1	- Capillary Vasodilation (1/1) - Microhemorrhage/Hemorrhage (1/1)
	**Hamaguchi 2021**	11**	- Microhemorrhage/Hemorrhage (11/11) - Bushy/Ramified Capillaries (1/11) - Irregularly Enlarged Capillaries (10/11) - Loss of Capillaries (6/11) - Disorganized Capillaries (1/11)
	**Alqatari 2018**	1	- Scleroderma Pattern (Early and Active Patterns) (1/1)
	**Molina-Ruiz 2015**	1*	- Large Capillary Loops (1/1) - Microhemorrhage/Hemorrhage (1/1) - Loss of Capillaries (1/1)
	**Fenando 2020**	1	- Dilated Capillary Loops (1/1) - Periungal Erythema (1/1) - Loss of Capillaries (1/1)
	**Kubo 2019**	16	- Scleroderma Spectrum Abnormalities (Subtype Unspecified) (14/16)
**Anti-TIF1-γ**	**Sugimoto 2021**	1	- Enlarged Capillaries (1/1) - Giant Capillaries (1/1) - Microhemorrhage/Hemorrhage (1/1)
	**Kubo 2019**	9	- Scleroderma Spectrum Abnormalities (Subtype Unspecified) (8/9)
**Anti-NXP2**	**Marchitto 2023**	1	- Dilated Capillary Loops (1/1) - Proximal Nail Fold Dystrophy [Samitz Sign] (1/1) - Loss Of Capillaries (1/1) - Ragged Cuticles (1/1)
	**Milne 2022**	1	- Dilated Vessels (1/1) - Loss of Capillaries (1/1)
	**Mugii 2023**	4**	- Irregularly Enlarged Capillaries (4/4) - Microhemorrhage/Hemorrhage (4/4) - Loss of Capillaries (4/4) - Avascular areas (1/4)
**Anti-Jo-1**	**Dandelooy 2016**	1	- Giant Capillaries (1/1) - Microhemorrhage/Hemorrhage (1/1) - Bushy/Ramified Capillaries (1/1) - Capillary Loss (1/1)
	**Riccieri 2010**	1	- Megacapillaries (1/1) - Microhemorrhage/Hemorrhage (1/1) - Ramified Capillaries (1/1) - Disorganized Capillaries (1/1) - Bizarre/Atypia (1/1) - Neoangiogenesis (1/1) - Avascular Areas (1/1)
**Anti-RO52**	**Molina-Ruiz 2015**	1*	- Large Capillary Loops (1/1) - Microhemorrhage/Hemorrhage (1/1) - Loss of Capillaries (1/1)
**Anti-Mi2**	**Pokhrel 2020**	1	- Variant and Simple Raynaud’s (1/1)

* This patient was both Anti-MDA5 and Anti-RO52 Autoantibody Positive

** This study included only patients who were positive for this antibody

## Discussion

Several studies have independently reported prevalence of microscopic nailfold features in patients with DM. In particular, the scleroderma spectrum pattern has been identified as the most prevalent nailfold capillaroscopic finding among patients with DM [48, 49]. However, previous studies focusing on microscopic findings in DM have limited sample sizes and do not explore associations with MSA/MAA. This review summarizes the microscopic nailfold findings in 346 DM patients across 37 studies and demonstrates the association between specific findings and MSA/MAA positivity.

The results of this study align with previous data indicating the scleroderma-spectrum pattern, as observed via nailfold microscopy, is prevalent amongst patients with DM [4, 7]. Furthermore, several of the most prevalent individual findings reported outside of an identifiable pattern are features commonly observed as part of the larger scleroderma spectrum pattern. In particular, the 1st, 2nd, 4th, and 5th most prevalent nailfold findings observed in this review (‘increase in vessel diameter’, ‘decreased vascularity’, ‘microhemorrhage/hemorrhage’, and ‘disturbed distribution/disorganized capillaries/architecture’, respectively) are features commonly included within the greater scleroderma-spectrum pattern. The scleroderma pattern is characteristically defined by enlargement and deformation of the capillary bed in association with capillary loss or avascularity, with variable inclusion of capillary budding and hemorrhage as criteria [9, 45]. The results of our review, therefore, bolster the evidence for the association of these nailfold capillaroscopic findings with DM.

Our review demonstrates a range of nailfold capillaroscopic findings in patients positive for MSAs but reveals a dearth of studies reporting MAAs in direct association with microscopic nailfold findings. However, several preliminary observations can be made. A previous cohort study performed by Torres-Ruiz et al. investigated the nailfold video capillaroscopic findings in patients with IIM, including 95 patients with DM. Although the study from Torres-Ruiz et al. did not fit the inclusion criteria for this review, their results reveal findings analogous to our own results. Torres-Ruiz et al. reported an elevated frequency of dilated capillaries in DM patients with anti-MDA-5 positivity relative to patients positive for other DM-associated antibodies [4]. Our results demonstrate the presence of vessel dilation or enlargement in 13 of the 31 (41.9%) patients who were positive for anti-MDA-5, further suggesting a potential association of this finding with anti-MDA-5 positivity. Likewise, Kubo et al. reported a statistically significant higher prevalence of positivity for anti-MDA-5 autoantibodies and anti-TIF-1γ autoantibodies in patients with NVC scleroderma spectrum abnormalities relative to those without NVC scleroderma spectrum abnormalities [10]. The results from our review, which included patients from Kubo et al., demonstrate numerous nailfold capillaroscopic abnormalities in patients positive for anti-MDA5 and anti-TIF1-γ, with both groups containing a number of individuals with scleroderma-spectrum abnormalities. Broadly, our findings suggest an array of features may be observed in patients positive for MSAs/MAAs. These results underscore the need for further elucidation of capillaroscopic and microscopic patterns potentially unique to antibody-associated DM. While existing studies compare relative frequencies of nailfold capillaroscopic findings in patients with MSAs, future, large cohort studies focused on the direct comparison of nailfold capillaroscopic findings to MSA and MAA status are needed.

The retrospective nature of this review precluded standardization of reporting and defining microscopic findings. In particular, increase in vessel diameter was often reported inconsistently, with the terms ‘enlarged’ or ‘large’ used variably to refer to dilated, mega/giant capillaries, or to refer to an increase in diameter without elucidation of actual diameter size. Additionally, definitions of the scleroderma-spectrum pattern and its subtypes varied or were not reported. Accordingly, utilization of standardized criteria for identification of vessel enlargement and reporting of scleroderma-spectrum pattern may lead to improved understanding of the true prevalence of nailfold capillaroscopic and microscopic findings observed in DM. A possible method of standardization may be consistent reporting of capillaries with a diameter > 20 μm as ‘dilated’, while ‘mega/giant capillaries’ may be defined as capillaries with a diameter > 50 µm [8]. Similarly, when possible, reporting the specific observed stage of scleroderma-spectrum pattern (‘early’, ‘active’ and/or ‘late’), according to the standards established by Cutolo et al., may lead to increased clarity regarding findings [48].

Finally, the scope of some included studies was limited to identification of pre-selected nailfold microscopic features, potentially leading to a disproportionate elevation in the reported prevalence of select features relative to others. Given the limited number of studies reporting microscopic nailfold features in direct association with DM, we believe the inclusion of these studies remains beneficial to the understanding of the nailfold features commonly observed on nailfold microscopic exam in patients with DM. However, we acknowledge the inclusion of these studies may introduce selection bias, and encourage future, large cohort studies to report all nailfold capillaroscopic and microscopic findings identified on examination.

Our study bolsters the current data pertaining to nailfold capillaroscopic findings in patients with DM, demonstrating that the scleroderma-spectrum pattern and associated individual features are the predominant nailfold capillaroscopic and microscopic findings across 37 studies. Furthermore, our study provides an overview of the data regarding capillaroscopic findings directly associated with antibody status in patients with DM, which may allow for expansion of current phenotypic associations with myositis antibodies and serve as the basis for future investigations regarding the association of autoantibodies and nailfold findings. Future studies may focus on further examination of the association of specific MSA and MAA antibodies and nailfold findings, with the aim of expanding the phenotypic criteria of antibody-associated DM.

## Electronic supplementary material

Below is the link to the electronic supplementary material.

Supplementary Material 1

## Data Availability

No datasets were generated or analysed during the current study.
