# Supplementary material for: Nailfold microscopy in adult-onset dermatomyositis in association with myositis antibodies
Source: Arch Dermatol Res. 2024 Nov 20;317(1):34. doi: 10.1007/s00403-024-03521-z (PMC11579119; doi:10.1007/s00403-024-03521-z)
Supplement: Supplementary file 1 — Supplementary Material 1 [file 403_2024_3521_MOESM1_ESM.docx]

**Article Title:** Nailfold Microscopy in Adult-Onset Dermatomyositis in Association with Myositis Antibodies

**Journal Name:** Archives of Dermatological Research

**Author Names:** Elizabeth M. Flatley, Dina Collins, Tess M. Lukowiak, Jason H. Miller

**Corresponding Author:**

Elizabeth M. Flatley

Robert Wood Johnson Medical School, Rutgers University, New Brunswick, New Jersey

Email: [emf180@rwjms.rutgers.edu](mailto:emf180@rwjms.rutgers.edu)

**Supplementary Table 1.** Nailfold Microscopic Findings of Included Studies

| **Citation** | **Total Number of Patients with DM** | **Capillaroscopic Findings (N/Gross Number of Patients in Study/Report)** |
| --- | --- | --- |
| Buchanan 1968 | 7 | - Microhemorrhage/Hemorrhage (3/7) - Normal/No Changes (2/7) - Capillary Loop Diameter > 28 µm (5/7) |
| Maricq 1976 | 3 | - Dilated Loops (3/3):   - "extremely* dilated loops confined to nailfold-distal phalanx" (1/3)   - "definitely** dilated loops on nailfold-distal phalanx and definitely dilated loops found on at least one site other than nailfold-distal phalanx" (1/3)   - "extremely* dilated loops on nailfold-distal phalanx and extremely dilated or definitely dilated loops found at one site other than nailfold-distal phalnx" (1/3)   * Extremely dilated = “capillaries increased several times in size and capillary loops distorted"  ** Definitely dilated = “loop increased in size, but loop form preserved” |
| Wong 1988 | 1 | - Enlarged Loops (1/1) - Bushy Capillaries (1/1) - Avascular Area (1) - Extravasates (1/1) |
| Ganczarczyk 1988 | 16 | - Enlarged Loop (9/16) - Avascular area (15/16) |
| Bergman 2003 | 11 | - Scleroderma-Dermatomyositis Pattern (7/11)   Normal/No Changes (4/11) |
| Ekmekci 2005 | 1 | - Dilated Capillary Loops (1/1) - Tortuous Capillary Loops (1/1)   Avascular Areas (1/1) |
| DeAngelis 2010 | 1 | - Loop Widening (1/1) - Elongation (1/1) - Ramifying Capillaries (1/1) - Vascular Neoformation (1/1) - Striking Shape Heterogeneity (1/1) - Architectural Derangement (1/1) - Rearrangement (1/1) - Loss (Disappearance) of Capillaries (1/1) |
| Riccieri 2010 | 1 | - Megacapillaries/Giant capillaries (1/1) - Microhemorrhage/Hemorrhage (1/1) - Ramified Capillaries (1/1) - Disorganized Capillaries ("The distribution and number of capillary loops with marked architectural derangement") (1/1) - Loss of capillaries (1/1) - Avascular areas (1/1) |
| Ohtsuka 2012 | 10 | - Capillary Dilation (7/10) - Microhemorrhage/Hemorrhage (8/10) - Avascular area (7/10) - Normal/No Changes (2/10) - Dilation & Bleeding Pattern (8/10) |
| Pretel 2013 | 1 | Telangiectasias (1/1) |
| Lambova 2013 | 2 | - Capillary Dilation (2/2) - Megacapillaries/Giant capillaries (2/2) - Elongation (1/2) - Microhemorrhage/Hemorrhage (2/2) - Neoangiogenesis (1/2) - Severe Disarrangement (2/2) - Loss of Capillaries (1/2) - Avascular Area (1/2) - Scleroderma-Like Pattern (2/2) |
| Molina-Ruiz 2015 | 1 | - Large Capillary Loops (1/1) - Microhemorrhage/Hemorrhage (1/1) - Loss of Capillaries (1/1) |
| Ring 2016 | 1 | - Capillary Dilation (1/1) - Disorganized Capillaries (1/1) - Avascular area (1/1) - Y-shaped vessel formation (1/1) |
| McBride 2016 | 1 | - Nailfold Telangiectasia (1)   Cuticular (Eponychial) Hemosiderin-Containing Deposits (1/1) |
| Dandelooy 2016 | 1 | - Megacapillaries/Giant capillaries (1/1) - Microhemorrhage/Hemorrhage (1/1) - Bushy Capillaries (1/1) - Loss of capillaries (1/1) |
| Faguer 2018 | 1 | Abnormal Tortuous (1/1) |
| Alqatari 2018 | 1 | - Early and Active Scleroderma Pattern (1/1) |
| Yang 2018 | 1 | - Capillary Dilation (1/1) - Microhemorrhage/Hemorrhage (1/1) - "Dendritic" Tortuosity (1/1) - Nail Fold Hyperkeratosis (1/1) |
| Kubo 2019 | 52 | Scleroderma Spectrum Overall (34/52):   - Early (10/52) - Active (5/52) - Late (2/52) - Scleroderma-Like (17/52) |
| Park 2019 | 1 | - Capillary Dilation (1/1) - Megacapillaries/Giant capillaries (1/1) - Tortuous Capillaries (1/1) - Avascular Area (1/1) - Abnormal (Decreased) Blood Flow (1/1) |
| Fenando 2020 | 1 | - Loop/Giant Loops (1/1) - Loss of capillaries (1/1) - Periungual Erythema (1/1) |
| Pokhrel 2020 | 1 | - Variant and Simple Raynaud's (1/1) |
| Hamaguchi 2021 | 11 | - Irregularly Enlarged Capillaries (10/11) - Microhemorrhage/Hemorrhage (11/11) - Bushy Capillaries (1/11) - Disorganization of the Vascular Array (1/11) - Loss of capillaries (6/11) |
| Sugimoto 2021 | 1 | - Enlarged Capillaries (1/1) - Giant Capillaries (1/1) - Microhemorrhage/Hemorrhage (1/1) |
| Monfort 2021 | 10 | - Enlarged Capillaries (1/10) - Megacapillaries/Giant Capillaries (5/10) - Elongation (1/10) - Microhemorrhage/Hemorrhage (8/10) - Bushy Capillaries (7/10) - Architectural Disorganization (7/10) - Tortuous Capillaries (2/10) - Loss of Capillaries (Capillary rarefaction 7/mm) (5/10) - Avascular Areas (4/10) - Scleroderma Spectrum Pattern (8/10)   Edema (4/10) |
| Milne 2022 | 1 | - Capillary Dilation (1/1) - Loss of capillaries (1/1) |
| Mentesoglu 2022 | 1 | - Loop/Giant Loops (1/1) - Microhemorrhage/Hemorrhage (1/1) |
| Shenavandeh 2022 | 106 | - Capillary Dilation (50/106) - Giant Loops (53/106) - Elongation (49/106) - Microhemorrhage/Hemorrhage (69/106) - Loss of capillaries (53/106) - Avascular Area (38/106) - Abnormal Blood Flow (16/106)   - Stasis (14/106)   - Thrombosis (2/106) - Nonspecific (26/106) - Scleroderma Pattern (78/106) - Disturbed Distribution (86); Normal/No Changes (2/106) - Unspecified abnormal shape (65/106) |
| Żychowska 2022 | 15 | - Capillary Dilation (8/15) - Giant Capillaries (4/15) - Elongation (10/15) - Microhemorrhage/Hemorrhage (8/15) - Bushy Capillaries (5/15) - Disorganized Capillaries (9/15) - Tortuous Capillaries (8/15) - Avascular Areas (9/15)   Subpapillary Plexus (8/15) |
| Asbeck 2022 | 1 | - Dilated Looped Telangiectasias (1/1) |
| Paudyal 2022 | 1 | - Telangiectasia (1/1) - Microhemorrhage/Hemorrhage (1/1) - Cuticular Dystrophy (1/1) - Periungual erythema (1/1) |
| Masson 2023 | 1 | - Megacapillaries/Giant capillaries (1/1) - Elongation (1/1) - Microhemorrhage/Hemorrhage (1/1) - Tortuosities (1/1) |
| Marchitto 2023 | 1 | - Capillary Dilation (1/1) - Loss of Capillaries (1/1) - Ragged Cuticles (1/1) - Proximal Nail Fold Dystrophy (Samitz Sign) (1/1) |
| Sugimoto 2023 | 1 | - Limited Vasodilation (1/1) - Microhemorrhage/Hemorrhage (1/1) |
| Ezeofor 2023 | 14 | - Capillary Dilation/Dilated Loops (5/14) - Loss of Capillaries (2/14) - Ragged Cuticles (3/14) - Capillary Pigmentation/Hyperpigmentation (1/14) - Cuticular Hypertrophy (1/14) - Normal/No Changes (8/14) |
| Paudyal 2023 | 62 | - Capillary Dilation (8/62) - Enlarged/Giant Capillaries (25/62) - Microhemorrhage/Hemorrhage (34/62) - Bushy Capillaries (47/62) - Crisscross Capillaries (20/62) - Avascular area (41/62) - Ragged Cuticles (38/62) - Dotted Vessel (2/62) - Brownish Pigmentation [Haem Deposits] (12/62) - Scleroderma-Like Pattern (26/62) - Hairpin Capillaries (10/62) - Periungal Erythema (24/62) |
| Mugii 2023 | 4 | - Irregularly Enlarged capillaries (4/4) - Microhemorrhage/Hemorrhage (4/4) - Reduced Number of Capillaries (4/4) - Avascular Areas [Moderate or Extensive Capillary Loss] (1/4) |
